# Assessment of *Plasmodium falciparum* anti-malarial drug resistance markers in *pfcrt* and *pfmdr1* genes in isolates from Honduras and Nicaragua, 2018–2021

**DOI:** 10.1186/s12936-021-03977-8

**Published:** 2021-12-14

**Authors:** Gustavo Fontecha, Alejandra Pinto, Osman Archaga, Sergio Betancourth, Lenin Escober, Jessica Henríquez, Hugo O. Valdivia, Alberto Montoya, Rosa Elena Mejía

**Affiliations:** 1 Microbiology Research Institute, National Autonomous University of Honduras, Tegucigalpa, Honduras; 2 National Malaria Laboratory, National Department of Surveillance, Ministry of Health of Honduras, Tegucigalpa, Honduras; 3 Department of Parasitology, U.S. Naval Medical Research Unit No, 6 (NAMRU-6), Lima, Peru; 4 National Center for Diagnosis and Reference, Health Ministry, Managua, Nicaragua; 5 Pan American Health Organization, Tegucigalpa, Honduras

**Keywords:** *Plasmodium falciparum*, Honduras, Nicaragua, *Pfcrt*, *Pfmdr*1, Drug resistance, Surveillance

## Abstract

**Background:**

Central America and the island of Hispaniola have set out to eliminate malaria by 2030. However, since 2014 a notable upturn in the number of cases has been reported in the Mosquitia region shared by Nicaragua and Honduras. In addition, the proportion of *Plasmodium falciparum* malaria cases has increased significantly relative to vivax malaria. Chloroquine continues to be the first-line drug to treat uncomplicated malaria in the region. The objective of this study was to evaluate the emergence of chloroquine resistant strains of *P. falciparum* using a genetic approach. *Plasmodium vivax* populations are not analysed in this study.

**Methods:**

205 blood samples from patients infected with *P. falciparum* between 2018 and 2021 were analysed. The *pfcrt* gene fragment encompassing codons 72–76 was analysed. Likewise, three fragments of the *pfmdr1* gene were analysed in 51 samples by nested PCR and sequencing.

**Results:**

All samples revealed the CVMNK wild phenotype for the *pfcrt* gene and the N86, Y184F, S1034C, N1042D, D1246 phenotype for the *pfmdr1* gene.

**Conclusions:**

The increase in falciparum malaria cases in Nicaragua and Honduras cannot be attributed to the emergence of chloroquine-resistant mutants. Other possibilities should be investigated further. This is the first study to report the genotype of *pfmdr1* for five loci of interest in Central America.

**Supplementary Information:**

The online version contains supplementary material available at 10.1186/s12936-021-03977-8.

## Background

The Americas reported more than 723,000 cases of malaria in 2019, which represents an increase of 7% compared to 2010. Venezuela (55%), Brazil (22%) and Colombia (11%) contributed with more than 86% of all malaria cases in the Americas [1]. In Central America both Belize and El Salvador are malaria free, and together with Haiti, Guatemala, and Honduras they have met the goal of the Global Technical Strategy 2016–2030 of reducing the incidence of cases by at least 40% [2]. In contrast, Costa Rica, the Dominican Republic, Nicaragua, and Panama have shown increases in the incidence of cases of more than 40% in 2020 [1].

**Fig. 1:**
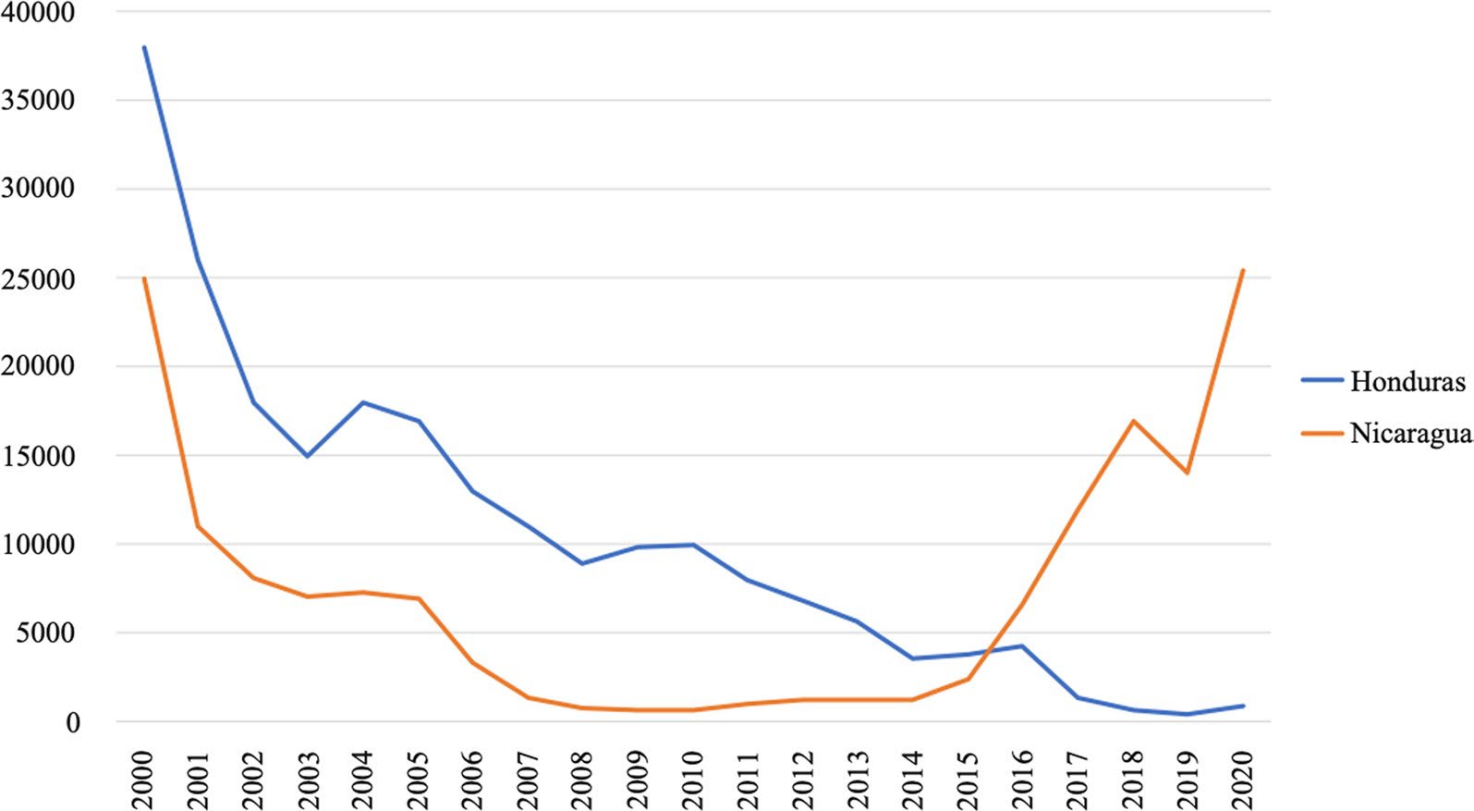
Number of malaria cases in Honduras and Nicaragua from 2000 to 2020. Malaria cases are diagnosed according to the national norm of each country and following the indications of the Pan American Health Organization

Central America contributes with only 2.4% of malaria cases in Latin America and the Caribbean. However, the Mosquitia region that includes Honduras and Nicaragua was responsible for almost 2% of cases in 2019 [1]. According to these data, the current achievements for malaria control in Central America are heterogeneous and, in some cases, a significant setback has been reported. For instance, Nicaragua reported more than 25,000 cases of malaria in 2020, surpassing the figures of 2000, which indicates two decades lost in progress towards the control and elimination of the disease (Fig. 1) (Personal communication by the National Center for Diagnosis and Reference, Health Ministry, Nicaragua). Another worrying data is the proportional increase in the number of cases of *Plasmodium falciparum* malaria in the Mosquitia. Malaria due to *P. falciparum* was less than 10% in 2008 in both countries, and in 2020 it exceeded 50% in Nicaragua and 29% in Honduras.

There are several hypotheses that try to explain this alarming situation. One of them is related to the possible appearance of resistance of the parasite against anti-malarial drugs. The countries of northern Central America and the island of Hispaniola are the only region in the world using chloroquine (CQ) as first-line of treatment against uncomplicated *P. falciparum* malaria [1]. The first cases of CQ resistance emerged in the early 1960s in Asia [3, 4], rendering it ineffective in almost all endemic territories in the tropics [5, 6]. Resistance to CQ is associated with mutations in the gene encoding the PfCRT protein (*P. falciparum* chloroquine resistance transporter) that is found on the membrane of the digestive vacuole (DV) of the parasite and transports 4-aminoquinoline drugs out of the vacuole [7]. Mutant resistant alleles of PfCRT prevent CQ from concentrating within acidic DV and preventing its toxic action [8]. Mutations in codons 72–76 of *pfcrt* are responsible for the resistant phenotype (CQR), generating five haplotypes associated with resistance: CVIET, SVMNT, SVIET, CVMNT and CVTNT [9, 10]. Despite the selective pressure exerted by the drug for decades, *P. falciparum* strains circulating in Central America have not developed resistance-associated mutations in the *pfcrt* gene [11–14] and they continue to hold the CVMNK susceptible wild genotype.

Along with *pfcrt*, although to a lesser degree, the *pfmdr1* gene has been associated with CQR, especially when the 86Y mutation appears in addition to the 76T mutation in *pfcrt* [15, 16]. *pfmdr1* gene has been less studied than *pfcrt* in *P. falciparum* strains circulating in Central America, revealing up to now the wild type at position N86 and a fixed mutation at position 184F [11, 14]. Mutations in other codons (1034, 1046 and 1246) reported to be associated with CQR have not been observed in *P. falciparum* strains in Central America.

Despite the lack of historical evidence of genetic mutations in the main markers of resistance to CQ in *P. falciparum* in the Central American Mosquitia, and the absence of anecdotal reports of therapeutic failure by physicians, this study aimed to support the active surveillance of recent genetic mutations to inform decision-makers about the potential emergence and spread of CQ-resistant strains in the region in the context of an increase in the number of cases especially in the Mosquitia.

## Methods

### Sample collection, ethics, and parasitological diagnosis

**Fig. 2:**
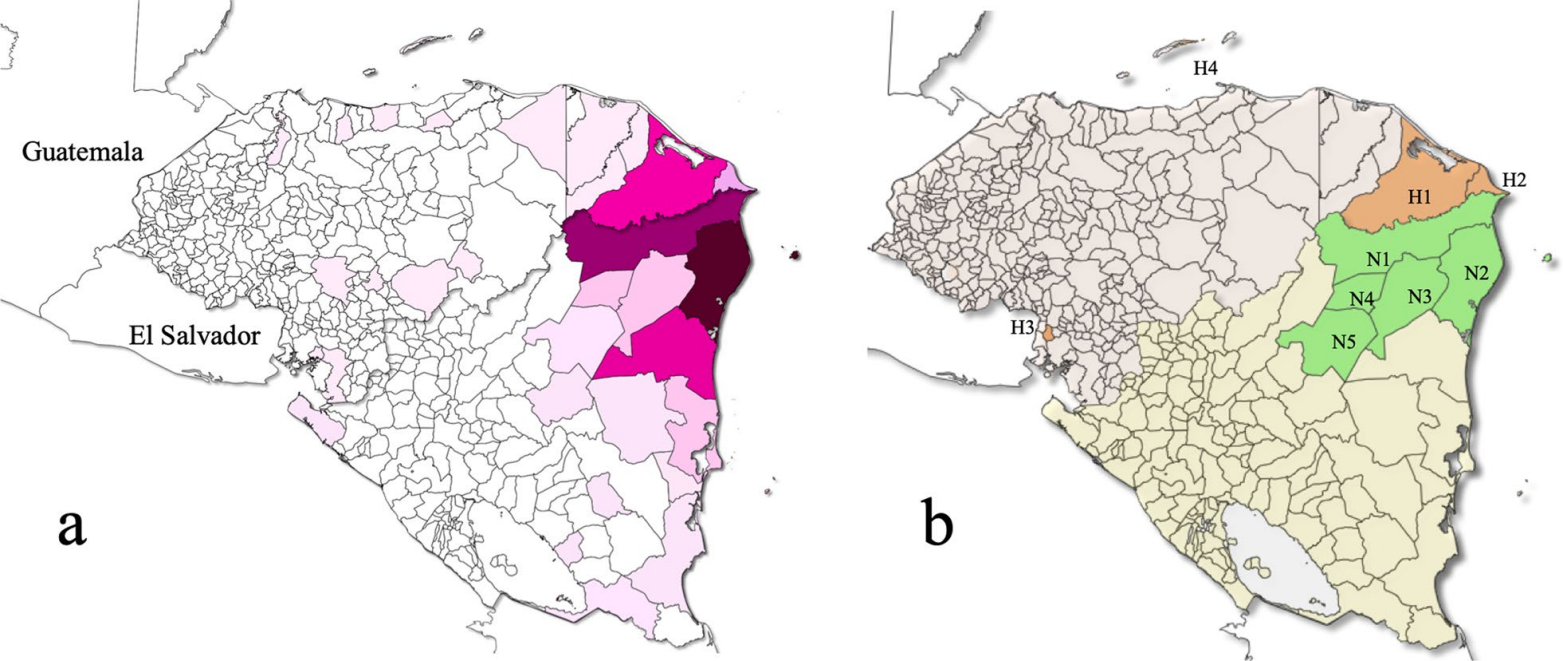
Map of Honduras and Nicaragua showing **a** the municipalities with reported malaria cases during 2020. The more intense the color, the greater the number of cases; and **b** the nine municipalities where the blood samples used in this study were collected. H1 = Puerto Lempira, H2 = Ramón Villeda Morales, H3 = Langue, H4 = José Santos Guardiola, N1 = Waspam, N2 = Puerto Cabezas, N3 = Rosita, N4 = Bonanza, N5 = Siuna, where H stands for Honduras and N for Nicaragua. Honduras is coloured in gray and Nicaragua in grayish yellow

This descriptive cross-sectional study included 205 blood samples from febrile patients diagnosed with malaria and who sought medical assistance in national sanitary facilities in four municipalities of Honduras (95 samples) and five municipalities of Nicaragua (110 samples) (Fig. 2).

Blood samples for molecular analysis were collected by fingerstick on Whatman FTA filter paper at the time of patient recruitment. In addition, blood samples were collected from febrile patients for the microscopic diagnosis of malaria by thick smear, before administering any anti-malarial treatment. The thick smear reading was performed by trained personnel according to the routine diagnostic protocols of both countries [17].

### DNA extraction and molecular confirmation of *P. falciparum* infections

DNA was extracted from blood on filter paper cards using a Chelex-100 based method (Bio-Rad Laboratories, Inc, EE. UU.) [18]. Microscopic diagnosis of the parasite species was confirmed by amplification of the 18S rRNA gene as described in Singh et al. [19].

### Amplification of *pfcrt* and *pfmdr1* gene fragments

A fragment of the *pfcrt* gene encompassing codons 72–76 was amplified by nested PCR. Briefly, for the first round of PCR, 10 µL of genomic DNA was used in a volume of 50 µL containing 25 µL of Taq Master Mix and 2 µL of each primer [13, 20] (Table 1) at a concentration of 10 µM and a remaining volume of nuclease free water. The reaction was mixed and subjected to the following program: initial denaturation at 94 °C for 10 min, followed by 35 cycles of 94 °C for 30 s, 59 °C for 30 s, and 72 °C for 30 s, and a final extension at 72 °C for 10 min. For the 2nd round of PCR, 2.0 μL of PCR products with 25.0 μL Taq Master Mix, 1.0 μL of each primer (10 μM), and _dd_H_2_O (up to 50.0 μl) were mixed and subjected to the following programme: initial denaturation at 94 °C for 10 min, followed by 30 cycles of 95 °C for 30 s, 56 °C for 30 s, and 72 °C for 30 s, and a final extension at 72 °C for 10 min.

**Table 1:** Sequence of primers used in nested PCR for the amplification of the *pfcrt* and *pfmdr1* genes

Gene	Primer	Sequence 5′- 3′	Size bp	References
*pfcrt*	AL6821	AGCAAAAATGACGAGCGTTATAG	559	[11]
	AL6822	ATTGGTAGGTGGAATAGATTCTC		
	AL5631	TTTTTCCCTTGTCGACCTTAAC	264	
	AL5632	AGGAATAAACAATAAAGAACATAATCATAC		
*pfmdr*1 SNPs 86, 184	MDR1-1F	TTAAATGTTTACCTGCACAACATAGAAAATT	612	[17]
	MDR1-1R	CTCCACAATAACTTGCAACAGTTCTTA		
	MDR1-2F	TGTATGTGCTGTATTATCAGGA	526	
	MDR1-2R	CTCTTCTATAATGGACATGGTA		
*pfmdr*1 SNPs 1034, 1046	1042-A	GTCGAAAAGACTATGAAACGTAGA	711	[17]
	1042-C	CTCAAATGATAATTTTGCAT		
	1042-B	GATCCAAGTTTTTTAATACA		
	1042-C	CTCAAATGATAATTTTGCAT		
*pfmdr*1 SNPs 1246	1246-A	GTGGAAAATCAACTTTTATGA	500	[17]
	1246-B	TTAGGTTCTCTTAATAATGCT		
	1246-C	GACTTGAAAAATGATCACATT	412	
	1246-D	GTCCACCTGATATGCTTTT		

In addition, three regions of the *pfmdr*1 gene were amplified to identify five polymorphisms at codons 86 and 184 (first segment), 1034 and 1046 (second segment), and 1246 (third segment) (Additional file 1: Fig. S1). The three regions were amplified by nested PCRs. In the first rounds of PCR, 2 µL of genomic DNA, 12.5 µL of Taq Master Mix, 1 µL of each primer (10 µM) (Table 1) and 8.5 µL of _dd_H_2_O were added. The second rounds of PCR used 2 μL of products from the first round, 25 μL of Taq Master Mix, 2 μL of each primer (10 μM), and 19 μL of _dd_H_2_O. The same amplification program was used for all reactions except the annealing temperature (52 °C for the first round and 54 °C for the second round): initial denaturation at 95 °C for 3 min, followed by 35 cycles of 95 °C for 30 s, annealing for 30 s, and 72 °C for 1 min, and a final extension at 72 °C for 5 min. PCR products were resolved on 2% agarose gels stained with ethidium bromide and visualized under UV transillumination.

### Sequence analysis

Purification and sequencing of gene fragments were carried out on both strands with their respective nested primers using a commercial service (Psomagen, Inc., Maryland, USA). Sequences were trimmed at both 5′ and 3′ ends with the Geneious^®^9.1.7 software and queried against international databases contained in the National Centre for Biotechnology Information (NCBI) to confirm the identity of the sequences. Subsequently, the sequences were analysed in search of target polymorphisms of interest for both genes. The sequences obtained were deposited in the NCBI database.

### Ethical considerations

The ethics committee (CEI-MEIZ) of the National Autonomous University of Honduras (UNAH) reviewed and approved the study under protocol number 03-2020. Consent to participate was waived for the following reasons: (a) No personal information was included. (b) The study was beneficial to public health and (c) does not harm the participants. Blood filter paper samples were collected for parasite species identification and analysis of genes associated with drug resistance, in accordance with national regulations and for routine malaria surveillance purposes.

## Results

In this study, 205 blood samples on filter paper collected for routine malaria diagnosis in Honduras and Nicaragua were analysed. None of the cases were imported from outside the region. Seven samples from 2018, 44 samples from 2019, 113 from 2020 and 41 samples from 2021 were analysed (Additional file 2: Table S1). Of 205 samples diagnosed by microscopic examination as *P. falciparum*, 201 were confirmed by PCR as *P. falciparum* and four samples (1.95%) were identified as mixed infections (*P. falciparum* and *P. vivax*). All samples were successfully amplified for the *pfcrt* gene fragment encompassing codons 72–76. Three segments of interest in the *pfmdr1* gene were amplified for 51 of the 205 samples (40 from Honduras and 11 from Nicaragua).

**Fig. 3:**
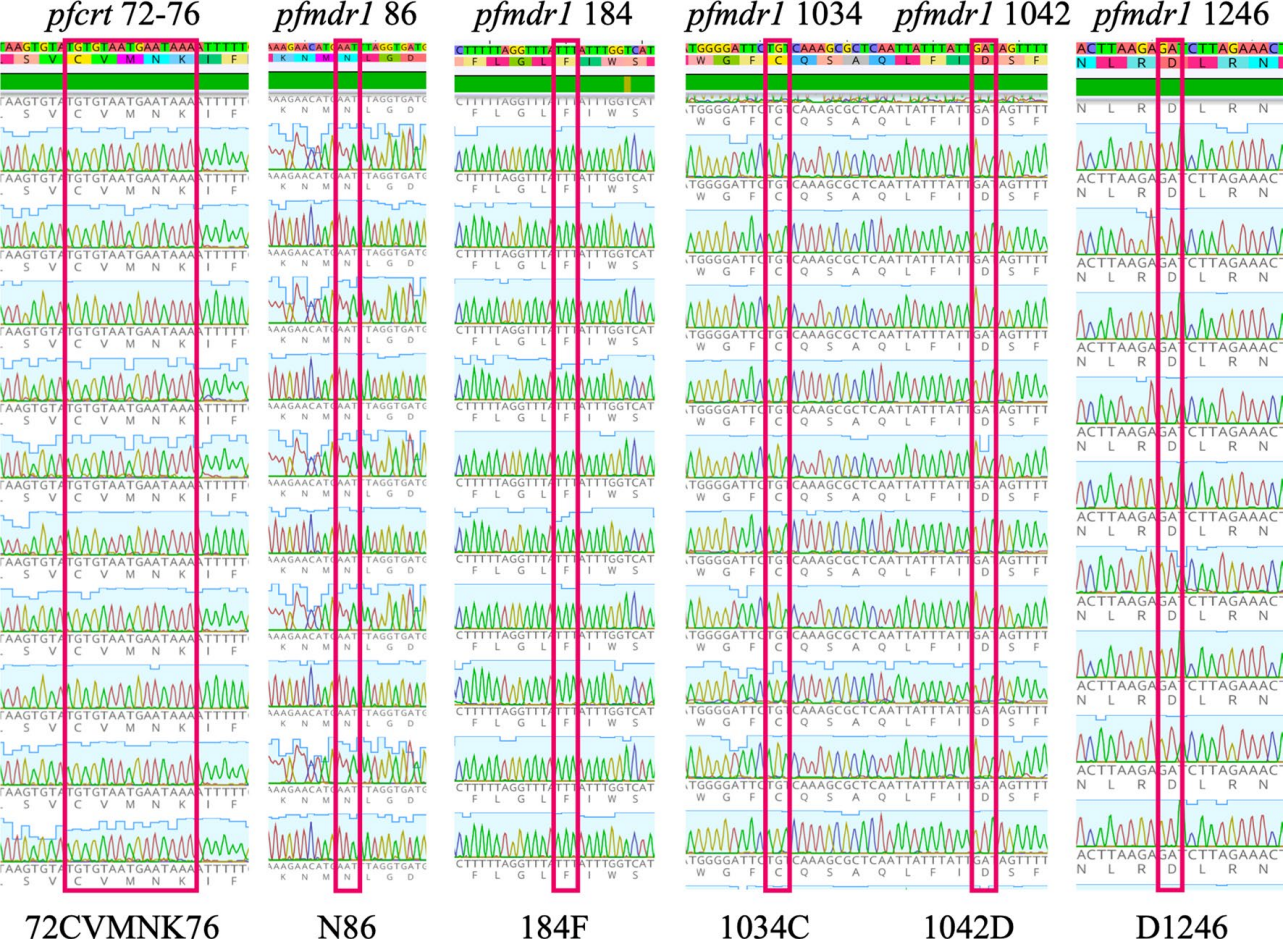
Chromatograms showing nucleotide and amino acid sequences from 6 codons associated with antimalarial resistance in the *pfcrt* and *pfmdr1* genes

All 205 samples were wild type for *pfcrt* (72-CVMNK-76) and thus sensitive to CQ. In the case of *pfmdr1*, all 51 samples presented the genotype NFCDD. Positions N86 and D1246 showed the wild type, while positions 184F, 1034C and 1042D showed a mutant phenotype in all samples (Fig. 3). Gene sequences were deposited in the NCBI database under the accession numbers MZ400792–MZ400875 (*pfcrt*) and MZ670132–MZ670296 (*pfmdr1*).

## Discussion

Chloroquine, one of the oldest synthetic drugs used in the treatment of malaria was developed in the 1930s, and became the most widely used synthetic antimalarial during the 1960s and 1970s [21]. However, the first chloroquine resistant (CQR) strains of *P. falciparum* began to be reported in Southeast Asia as early as 1957 [4]. CQR rapidly spread to sub-Saharan Africa and new resistant variants appeared in South America and Asia during the following decades [7, 22]. Surprisingly, northern Central America and the island of Hispaniola in the Caribbean are the only region in the world where *P. falciparum* strains are still susceptible to chloroquine (CQS) [12, 23]. Therefore, there are six countries in the Americas that still use chloroquine and primaquine as first-line of treatment against uncomplicated *P. falciparum* malaria: Guatemala, Honduras, Nicaragua, Costa Rica, Haiti, and the Dominican Republic [1]. Given the imminent threat of the spontaneous appearance of mutant parasite strains due to drug exerted pressure, as well as increasing human migration to North America through the Central American isthmus [24, 25], it is important to periodically monitor parasite populations for evidence of CQR [26]. This study is an effort to contribute to the routine surveillance of CQR in *P. falciparum* that has been carried out in Central America since 2010.

The *pfcrt* gene region spanning codons 72–76 in 205 *P. falciparum* isolates collected between 2018 and 2021 from Honduras and Nicaragua were sequenced. All samples showed a wild-type haplotype CVMNK associated with susceptibility to CQ. This result is consistent with previously published studies. The first study carried out with 30 samples collected in five departments of Honduras between 2004 and 2009 showed 100% of isolates with wild genotype, except for two individuals whose infection had been contracted in Asia and Africa [14]. A study of the therapeutic efficacy of CQ included 68 samples collected in Puerto Lempira, in the Honduran Mosquitia, revealing only wild genotypes in the *pfcrt* gene [13]. Likewise, an efficacy trial conducted in the North Atlantic Region (RAAN) of Nicaragua during 2005 and with samples from a surveillance study from 2011 showed that 96 of 98 samples had the CVMNK phenotype, while two samples had the CVIET CQR phenotype. Unfortunately, the authors were unable to confirm whether these cases were imported or not [27]. In a 2014 publication, the authors evaluated 160 samples collected from several municipalities in Honduras between 2010 and 2013 and showing 100% wild genotypes of *pfcrt* [12]. Unpublished data revealed that 16 samples collected in 2015 from Guatemala also showed the wild genotype. In a more recent study including 16 specimens that underwent next generation sequencing, 3 specimens were found with a CQR haplotype SVMNT. The authors indicate that two of these samples were imported cases from Africa and that the third was a local case collected in 2013 in the Choluteca region [11]. However, no epidemiological information is offered on the case that can ensure that it was indeed a local case or a foreign migrant passing through the country.

In the case of Central America and Hispaniola, where circulating parasites still show a wild CQS genotype the presence of imported cases from countries with CQR strains [11, 27–34] puts at risk the efforts made in recent decades to achieve malaria elimination [2]. The continued finding of CQ susceptibility in recent years, indicates that the notable increase in malaria cases in Nicaragua from 2014 to the present (Fig. 1) is not caused by the appearance of CQR strains but to other phenomena that exceed the purposes of this study and must be promptly analysed in an integrated way.

The second gene analysed in this study was *pfmdr1*, a transporter on the membrane of the digestive vacuole that mediates the transfer of antimalarial drugs from the cytosol to the vacuole [35]. At least 5 SNPs have been described in *pfmdr1* presumably associated with resistance to different antimalarial drugs [36–38]. Unlike *pfcrt*, the influence of the *pfmdr1* gene on *P. falciparum* CQR is still not entirely clear, and the response to anti-malarials is likely to be a multigenic phenomenon that is affected by the sum of mutations in different transporter genes [39]. To shed some light on this topic, three fragments of the *pfmdr1* gene encompassing codons 86, 184, 1034, 1042, and 1246 in 51 samples that had shown a wild type *pfcrt* genotype were sequenced. All samples showed an NFCDD haplotype, with wild type alleles at positions 86 and 1246, and mutant alleles at 184, 1034, and 1042. There are two published studies on this gene in samples from Honduras that coincide with the results of this study. A first study in 2011 showed that all 30 samples tested had the wild N86 genotype [14]. Similarly, a second study revealed the genotype N86, 184F, D1246 in all 16 samples [11]. This is the first study to analyse the five codons of interest in the *pfmdr1* gene in samples from Honduras and Nicaragua.

A study carried out in Haiti found the haplotype N86/184F in 108 samples analysed after the 2010 earthquake [30]. A second study carried out in Haiti amplified 54 samples and analysed all five codons, finding mutations only in codon 184 (NFSND) [34]. Likewise, six patients infected by *P. falciparum* in Punta Cana in the Dominican Republic were described as carriers of the 184F mutation [33]. On the other hand, there are several studies that reported different haplotypes of *pfmdr1* in South America. As in the present study, all reported exclusively the wild-type N86 [40–45] that seems to be a common characteristic in the strains of the continent. Some authors propose that CQR mediated by *pfcrt* mutations is modulated somehow by mutations in *pfmdr1*, and that the mutant alleles 86Y and 184F are the most relevant [46, 47]. According to the literature all the *P. falciparum* isolates show the 184F mutant allele in Central America [11], Haiti [30, 34] and South America [41–45, 48–50]. Mutation 184F is believed to have a limited effect in the absence of a mutation at codon 86 [51, 52]. Consequently, a *pfcrt* haplotype CVMNK together with a wild type N86 allele in most parasite strains in Central America and Haiti (despite the presence of 184F mutants), would allow predicting that it is unlikely that short-term CQR will appear in the region due to an accumulation of mutations in both genes.

Codon 1034 is more heterogeneous on the American continent. Both the wild genotype S1034, and the mutant 1034C, have been described in Colombia, Venezuela, Peru, and Suriname. The wild genotypes 1042D and 1246Y are the most frequent in the region [41, 42, 44, 45, 50, 53–56]. The NFCDD signature found in this study in all samples analysed has also been described in Peru (94.5–100%) [42, 45], French Guiana (0.2%) [56], Ghana (43.5%) [57], and Yemen (57%) [58]. It is complex to interpret the role that mutations in *pfmdr1* play on the modulation of resistance to the different antimalarial drugs available. However, in the absence of mutations in *pfcrt* in parasites from Honduras and Nicaragua, where CQ remains the first-line of treatment for uncomplicated malaria, mutant haplotypes in *pfmdr1* do not appear to be an important variable to consider at present.

Some limitations of the study are a relatively low number of samples analysed, the exclusion of *P. vivax*, which continues to be the predominant species of the parasite in the region, and the lack of clinical data on the susceptibility of the parasite to chloroquine, that could only be obtained from an in vivo efficacy study.

## Conclusions

This study established that there are still no mutations linked to antimalarial resistance in the *pfcrt* gene in *P. falciparum* isolates from Honduras and Nicaragua. Furthermore, the predominant haplotype for five codons of interest of *pfmdr1* gene is reported for the first time, revealing three positions with fixed mutations. The increasing number of malaria cases reported in Nicaragua since 2014 cannot be attributed to the emergence of resistance to CQ in *P. falciparum*. The evidence obtained in this study supports the hypothesis that CQ remains an effective drug for the treatment of uncomplicated *P. falciparum* malaria in the Mosquitia, although an in vivo efficacy study with chloroquine is necessary as definitive evidence.

## Supplementary Information

**Additional file 1: Fig. S1**. Scheme of the genes *pfcrt* (a) and *pfmdr1* (b-d) showing the names and targets of the primers, sizes of the amplicons, and location of the polymorphisms of interest.

**Additional file 2: Table S1.** Complete database with sequencing results for *pfcrt*, *pfmdr1*, and geographic origin of the samples.

## Data Availability

All data generated or analysed during this study are included in this published article.

## Abbreviations

*pfcrt*: *Plasmodium falciparum* Chloroquine resistance transporter
*pfmdr*1: *Plasmodium falciparum* Multidrug resistance 1
CQ: Chloroquine
CQR: Chloroquine resistant
CQS: Chloroquine susceptible
SNP: Single nucleotide polymorphism
RAAN: Autonomous North Atlantic Region of Nicaragua

## References

[1] WHO. World malaria report 2020: 20 years of global progress and challenges. Geneva: World Health Organization, 2020.

[2] WHO. Global technical strategy for malaria 2016–2030. Geneva: World Health Organization, 2015.

[3] White NJ (2004). Antimalarial drug resistance. J Clin Invest.

[4] Harinasuta T, Suntharasamai P, Viravan C (1965). Chloroquine-resistant falciparum malaria in Thailand. Lancet.

[5] Wongsrichanalai C, Pickard AL, Wernsdorfer WH, Meshnick SR (2002). Epidemiology of drug-resistant malaria. Lancet Infect Dis.

[6] Talisuna AO, Bloland P, D'Alessandro U (2004). History, dynamics, and public health importance of malaria parasite resistance. Clin Microbiol Rev.

[7] Wicht KJ, Mok S, Fidock DA (2020). Molecular mechanisms of drug resistance in *Plasmodium falciparum* malaria. Annu Rev Microbiol.

[8] Ecker A, Lehane AM, Clain J, Fidock DA (2012). PfCRT and its role in antimalarial drug resistance. Trends Parasitol.

[9] Awasthi G, Satya Prasad GB, Das A (2012). Pfcrt haplotypes and the evolutionary history of chloroquine-resistant *Plasmodium falciparum*. Mem Inst Oswaldo Cruz.

[10] Sidhu AB, Verdier-Pinard D, Fidock DA (2002). Chloroquine resistance in *Plasmodium falciparum* malaria parasites conferred by pfcrt mutations. Science.

[11] Valdivia HO, Villena FE, Lizewski SE, Garcia J, Alger J, Bishop DK (2020). Genomic surveillance of *Plasmodium falciparum* and *Plasmodium vivax* cases at the University Hospital in Tegucigalpa. Honduras Sci Rep.

[12] Fontecha GA, Sanchez AL, Mendoza M, Banegas E, Mejia-Torres RE (2014). A four-year surveillance program for detection of *Plasmodium falciparum* chloroquine resistance in Honduras. Mem Inst Oswaldo Cruz.

[13] Mejia Torres RE, Banegas EI, Mendoza M, Diaz C, Bucheli ST, Fontecha GA (2013). Efficacy of chloroquine for the treatment of uncomplicated *Plasmodium falciparum* malaria in Honduras. Am J Trop Med Hyg.

[14] Jovel IT, Mejia RE, Banegas E, Piedade R, Alger J, Fontecha G (2011). Drug resistance associated genetic polymorphisms in *Plasmodium falciparum* and *Plasmodium vivax* collected in Honduras Central America. Malar J.

[15] Duraisingh MT, Cowman AF (2005). Contribution of the pfmdr1 gene to antimalarial drug-resistance. Acta Trop.

[16] Picot S, Olliaro P, de Monbrison F, Bienvenu AL, Price RN, Ringwald P (2009). A systematic review and meta-analysis of evidence for correlation between molecular markers of parasite resistance and treatment outcome in falciparum malaria. Malar J.

[17] External quality assurance program for malaria microscopy diagnosis https://www.paho.org/hq/dmdocuments/2013/PEED-Malaria-OPS-Eng-2011-1.pdf.

[18] de Lamballerie X, Zandotti C, Vignoli C, Bollet C, de Micco P (1992). A one-step microbial DNA extraction method using “Chelex 100” suitable for gene amplification. Res Microbiol.

[19] Singh B, Bobogare A, Cox-Singh J, Snounou G, Abdullah MS, Rahman HA (1999). A genus- and species-specific nested polymerase chain reaction malaria detection assay for epidemiologic studies. Am J Trop Med Hyg.

[20] She D, Wang Z, Liang Q, Lu L, Huang Y, Zhang K (2020). Polymorphisms of pfcrt, pfmdr1, and K13-propeller genes in imported falciparum malaria isolates from Africa in Guizhou province. China BMC Infect Dis.

[21] Packard RM (2014). The origins of antimalarial-drug resistance. N Engl J Med.

[22] Maberti S (1994). Milestones in parasitology (1). Parasitol Today.

[23] Vincent JP, Komaki-Yasuda K, Existe AV, Boncy J, Kano S (2018). No *Plasmodium falciparum* chloroquine resistance transporter and artemisinin resistance mutations. Haiti Emerg Infect Dis.

[24] Boggild AK, Geduld J, Libman M, Ward BJ, McCarthy AE, Doyle PW (2014). Travel-acquired infections and illnesses in Canadians: surveillance report from CanTravNet surveillance data, 2009–2011. Open Med.

[25] Harvey K, Esposito DH, Han P, Kozarsky P, Freedman DO, Plier DA (2013). Surveillance for travel-related disease–GeoSentinel Surveillance System, United States, 1997–2011. MMWR Surveill Summ.

[26] Hamre KES, Pierre B, Namuyinga R, Mace K, Rogier EW, Udhayakumar V (2020). Establishing a national molecular surveillance program for the detection of *Plasmodium falciparum* markers of resistance to antimalarial drugs in Haiti. Am J Trop Med Hyg.

[27] Sridaran S, Rodriguez B, Soto AM, Macedo De Oliveira A, Udhayakumar V (2014). Molecular analysis of chloroquine and sulfadoxine-pyrimethamine resistance-associated alleles in *Plasmodium falciparum* isolates from Nicaragua. Am J Trop Med Hyg.

[28] Londono BL, Eisele TP, Keating J, Bennett A, Chattopadhyay C, Heyliger G (2009). Chloroquine-resistant haplotype *Plasmodium falciparum* parasites. Haiti Emerg Infect Dis.

[29] Gharbi M, Pillai DR, Lau R, Hubert V, Khairnar K, Existe A (2012). Chloroquine-resistant malaria in travelers returning from Haiti after 2010 earthquake. Emerg Infect Dis.

[30] Morton LC, Huber C, Okoth SA, Griffing S, Lucchi N, Ljolje D (2016). *Plasmodium falciparum* drug-resistant haplotypes and population structure in postearthquake Haiti, 2010. Am J Trop Med Hyg.

[31] Obaldia N, Baro NK, Calzada JE, Santamaria AM, Daniels R, Wong W (2015). Clonal outbreak of *Plasmodium falciparum* infection in eastern Panama. J Infect Dis.

[32] Juliao PC, Sosa S, Gonzalez LD, Padilla N, Ortiz L, Goldman I (2013). Importation of chloroquine-resistant *Plasmodium falciparum* by Guatemalan peacekeepers returning from the Democratic Republic of the Congo. Malar J.

[33] Chenet SM, Silva-Flannery L, Lucchi NW, Dragan L, Dirlikov E, Mace K (2017). Molecular characterization of a cluster of imported malaria cases in Puerto Rico. Am J Trop Med Hyg.

[34] Elbadry MA, Existe A, Victor YS, Memnon G, Fukuda M, Dame JB (2013). Survey of *Plasmodium falciparum* multidrug resistance-1 and chloroquine resistance transporter alleles in Haiti. Malar J.

[35] Ibraheem ZO, Abd Majid R, Noor SM, Sedik HM, Basir R (2014). Role of different pfcrt and pfmdr-1 mutations in conferring resistance to antimalaria drugs in *Plasmodium falciparum*. Malar Res Treat..

[36] Reed MB, Saliba KJ, Caruana SR, Kirk K, Cowman AF (2000). Pgh1 modulates sensitivity and resistance to multiple antimalarials in *Plasmodium falciparum*. Nature.

[37] Sidhu AB, Valderramos SG, Fidock DA (2005). pfmdr1 mutations contribute to quinine resistance and enhance mefloquine and artemisinin sensitivity in *Plasmodium falciparum*. Mol Microbiol.

[38] Babiker HA, Pringle SJ, Abdel-Muhsin A, Mackinnon M, Hunt P, Walliker D (2001). High-level chloroquine resistance in Sudanese isolates of *Plasmodium falciparum* is associated with mutations in the chloroquine resistance transporter gene pfcrt and the multidrug resistance gene pfmdr1. J Infect Dis.

[39] Mu J, Ferdig MT, Feng X, Joy DA, Duan J, Furuya T (2003). Multiple transporters associated with malaria parasite responses to chloroquine and quinine. Mol Microbiol.

[40] Restrepo-Pineda E, Arango E, Maestre A, Do Rosario VE, Cravo P (2008). Studies on antimalarial drug susceptibility in Colombia, in relation to Pfmdr1 and Pfcrt. Parasitology.

[41] Escobar DF, Lucchi NW, Abdallah R, Valenzuela MT, Udhayakumar V, Jercic MI (2020). Molecular and epidemiological characterization of imported malaria cases in Chile. Malar J.

[42] Bacon DJ, McCollum AM, Griffing SM, Salas C, Soberon V, Santolalla M (2009). Dynamics of malaria drug resistance patterns in the Amazon basin region following changes in Peruvian national treatment policy for uncomplicated malaria. Antimicrob Agents Chemother.

[43] Labadie-Bracho M, Adhin MR (2013). Increased pfmdr1 copy number in *Plasmodium falciparum* isolates from Suriname. Trop Med Int Health.

[44] Montenegro LM, de Las SB, Neal AT, Tobon-Castano A, Fairhurst RM, Lopera-Mesa TM (2021). State of artemisinin and partner drug susceptibility in *Plasmodium falciparum* clinical isolates from Colombia. Am J Trop Med Hyg.

[45] Huaman MC, Roncal N, Nakazawa S, Long TT, Gerena L, Garcia C (2004). Polymorphism of the *Plasmodium falciparum* multidrug resistance and chloroquine resistance transporter genes and in vitro susceptibility to aminoquinolines in isolates from the Peruvian Amazon. Am J Trop Med Hyg.

[46] Antony HA, Das S, Parija SC, Padhi S (2016). Sequence analysis of pfcrt and pfmdr1 genes and its association with chloroquine resistance in Southeast Indian *Plasmodium falciparum* isolates. Genom Data.

[47] Gupta H, Macete E, Bulo H, Salvador C, Warsame M, Carvalho E (2018). Drug-resistant polymorphisms and copy numbers in *Plasmodium falciparum*, Mozambique, 2015. Emerg Infect Dis.

[48] Aponte S, Guerra AP, Alvarez-Larrotta C, Bernal SD, Restrepo C, Gonzalez C (2017). Baseline in vivo, ex vivo and molecular responses of *Plasmodium falciparum* to artemether and lumefantrine in three endemic zones for malaria in Colombia. Trans R Soc Trop Med Hyg.

[49] Valenzuela G, Castro LE, Valencia-Zamora J, Vera-Arias CA, Rohrbach P, Saenz FE (2019). Genotypes and phenotypes of resistance in Ecuadorian *Plasmodium falciparum*. Malar J.

[50] Griffing S, Syphard L, Sridaran S, McCollum AM, Mixson-Hayden T, Vinayak S (2010). pfmdr1 amplification and fixation of pfcrt chloroquine resistance alleles in *Plasmodium falciparum* in Venezuela. Antimicrob Agents Chemother.

[51] Veiga MI, Dhingra SK, Henrich PP, Straimer J, Gnadig N, Uhlemann AC (2016). Globally prevalent PfMDR1 mutations modulate *Plasmodium falciparum* susceptibility to artemisinin-based combination therapies. Nat Commun.

[52] Sisowath C, Ferreira PE, Bustamante LY, Dahlstrom S, Martensson A, Bjorkman A (2007). The role of pfmdr1 in *Plasmodium falciparum* tolerance to artemether-lumefantrine in Africa. Trop Med Int Health.

[53] Baldeviano GC, Okoth SA, Arrospide N, Gonzalez RV, Sanchez JF, Macedo S (2015). Molecular epidemiology of *Plasmodium falciparum* malaria outbreak, Tumbes, Peru, 2010–2012. Emerg Infect Dis.

[54] Adhin MR, Labadie-Bracho M, Vreden S (2014). Gold mining areas in Suriname: reservoirs of malaria resistance?. Infect Drug Resist.

[55] Pillai DR, Hijar G, Montoya Y, Marouino W, Ruebush TK, Wongsrichanalai C (2003). Lack of prediction of mefloquine and mefloquine-artesunate treatment outcome by mutations in the *Plasmodium falciparum* multidrug resistance 1 (pfmdr1) gene for *P. falciparum* malaria in Peru. Am J Trop Med Hyg.

[56] Pelleau S, Moss EL, Dhingra SK, Volney B, Casteras J, Gabryszewski SJ (2015). Adaptive evolution of malaria parasites in French Guiana: reversal of chloroquine resistance by acquisition of a mutation in pfcrt. Proc Natl Acad Sci USA.

[57] Asare KK, Boampong JN, Duah NO, Afoakwah R, Sehgal R, Quashie NB (2017). Synergism between Pfcrt and Pfmdr1 genes could account for the slow recovery of chloroquine sensitive *Plasmodium falciparum* strains in Ghana after chloroquine withdrawal. J Infect Public Health.

[58] Al-Hamidhi S, Mahdy MA, Al-Hashami Z, Al-Farsi H, Al-Mekhlafi AM, Idris MA (2013). Genetic diversity of *Plasmodium falciparum* and distribution of drug resistance haplotypes in Yemen. Malar J.

