# Supplementary figures and images for: Assessment of Plasmodium falciparum anti-malarial drug resistance markers in pfcrt and pfmdr1 genes in isolates from Honduras and Nicaragua, 2018–2021
Source: Malar J. 2021 Dec 14;20:465. doi: 10.1186/s12936-021-03977-8 (PMC8670165; doi:10.1186/s12936-021-03977-8)

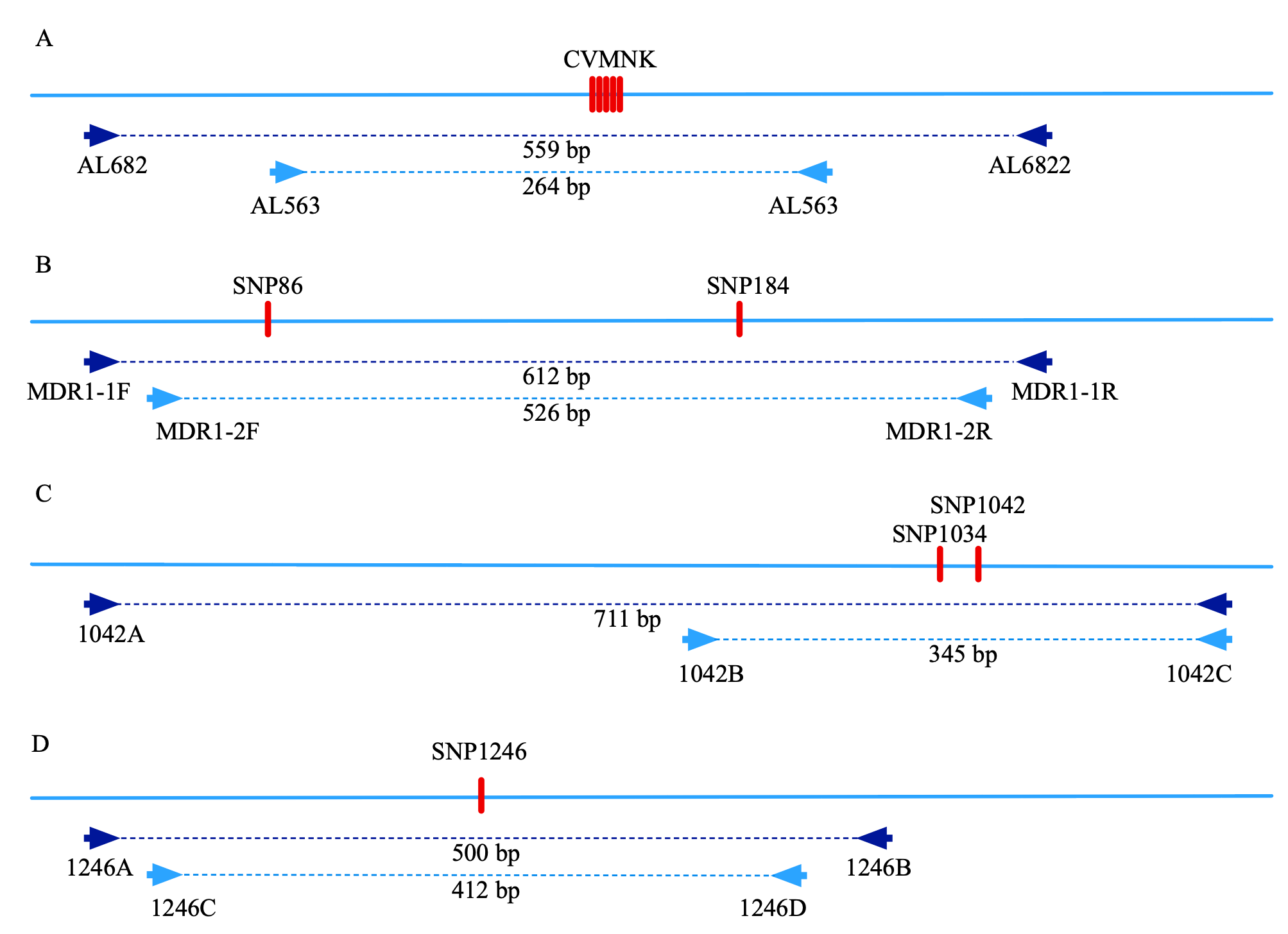

Supplement: Supplementary file 1 — Additional file 1: Fig. S1. Scheme of the genes pfcrt (a) and pfmdr1 (b-d) showing the names and targets of the primers, sizes of the amplicons, and location of the polymorphisms of interest. [file 12936_2021_3977_MOESM1_ESM.png]
